# Case of Diabetes, with an Account of the Appearances after Death, Stated in a Letter to Dr. Rollo

**Published:** 1799-10

**Authors:** Alexander Marcet

**Affiliations:** Member of the Royal College of Physicians, London; and Physician to the City Dispensary


### *Case of Diabetes, with an Account of the Appearances after Death, stated in a Letter to Dr. Rollo; by* ALEXANDER MARCET, M. D. *Member of the Royal College of Physicians, London; and Physician to the City Dispensary.*

DEAR SIR,

I WAS very much disappointed by not seeing you at the examination of the body of my diabetic patient, which took place on the 6th of the last month, according to the notice I had sent you. Dr. WILLAN and Dr. DELARIVE were present at the dissection; and Dr. MARSHALL was so obliging as to perform it. I am sensible how much more interesting it would have been to you if received from the hand of that experienced observer, and flatter myself he may yet be induced to give it to the public. In the mean time, I transmit, in compliance with your request, a short account of the case, and will afterwards relate, as well as I am able, the principal circumstances which presented themselves, or were pointed out to me, during and after the dissection.

The history of the case contains nothing, I believe, that has not been before observed in diabetes: but, as you will see from the account of the dissection, this patient also laboured under phthisis pulmonalis, a circumstance which I was not aware of, and which some other medical men who visited him with me at different periods, seemed to have likewise overlooked.

R. K. of Chancery-lane, a carpenter, aged 55, applied to the dispensary in Carey-street, in March, 1798. I then attended the dispensary as an assistant physician to Dr. Willan, and this man became one of my patients. He complained of great weakness and emaciation; of pains in his loins, and across his stomach; and of a slight cough, to which he had been at times subject, but which, he said, was but very trifling. He complained also of heat in his inside; but he never spit any blood; and his expectoration was never copious nor purulent. His tongue was dry, but clean and florid; his skin uncommonly dry, and had been so for a long time; his pulse was a little more frequent than natural. Those symptoms would have naturally led to the suspicion of consumption, and indeed it was the first idea that occurred to me; but having inquired more particularly into the circumstances of his illness, and having found he was labouring under diabetes, I thought I could explain the symptoms of pain, heat, and emaciation, without any other supposition, and I lost sight of the phthisical symptoms.

 I learnt

I learnt that his appetite was uſually very keen, though not ſo at that moment, owing, as he thought, to his having caught cold. His thirſt, however, was immoderate, and his urine, which was much more copious than natural, had the peculiar diabetic colour and properties. He did not paſs his water without being conſcious of it, but could not retain it a ſingle moment whenever he had a call to diſcharge it. His ancles frequently ſwelled towards evening, and his feet were in general cold; but he complained of occaſional heat in the palms of his hands. He had but a few teeth left, which were quite looſe in their ſockets. His ſpirits were extremely low, and had been ſo during the whole courſe of his illneſs; he was ſo deſpondent about nine months ago, as to make an attempt againſt his own life, which proved very nearly ſucceſsful. His memory ſeemed to be conſiderably impaired; and he could not diſtinctly remember in what manner he was firſt taken ill; but his wife told me his complaints had begun about eighteen months before; and that the firſt ſymptoms ſhe could trace, were an uncommon appetite and a proportionate thirſt. He had been a hard drinker all his life, and ſtill indulged the ſame habit. He had been, at different periods, ſubject to diarrhœa, but latterly complained of obſtinate coſtiveneſs. The quantity of his drink amounted to ſeven or eight pounds of beer, or ſpirits and water, in twenty-four hours; and he paſſed in a ſimilar ſpace of time, between four and ſix pounds of ſweet, pale urine, which yielded an uncommonly copious ſaccharine ſediment. According to the account of his wife, both his drink and urine were ſome time before much more conſiderable, and ſhe thought the quantity of his urine had been, at times, fully equal to that of his drink.

Theſe were the principal circumſtances of the caſe, of which I kept memorandums ever ſince I undertook the treatment of it; and though there have been within theſe fifteen months ſome fluctuations in the ſymptoms, I did not perceive any remarkable change in the general ſtate of the patient till a few weeks before his death. His legs and ancles then ſwelled very much; his pulſe became quicker, though not very weak nor irregular; his powers of digeſtion almoſt entirely ceaſed: frequent purging and vomiting ſupervened, which continued with little abatement to the moment of his death. He remained ſenſible till within a few hours before he expired, but he then loſt his power of ſpeech, and was ſoon after carried off in a fit of convulſion.

I ſaw him, for the laſt time, four days before his death. Till then, the diabetic ſymptoms had continued, and his urine had the qualities peculiar to the diſeaſe.

The method of treatment employed could ſcarcely afford any intereſting obſervation, ſince from the complicated and advanced ſtate of the diſeaſe, I could

I could only ufe palliative remedies. His ftomach was totally unable to bear the animal regimen, and there were but very few articles of food that could at all agree with him. He was very whimfical in his diet, and refufed, at times, to take any kind of animal food, whilft, unfortunately, he was very fond of fugar and fweet things of any kind. Opium always relieved him, and was repeatedly adminiftered in different forms. When he was coftive, aloes agreed with him well, and were often prefcribed. At a later period of the diforder, his bowels were in a very relaxed ftate, which could only be corrected by reftringents, either alone or combined with opiates. During the laft fummer he went to fpend a few months in the country, when he difcontinued the ufe of any medicines; he thought at firft the country air was of fome fervice to him, but at his return, he feemed to be nearly in the fame ftate as before.

*Diffection about thirty-fix Hours after Death.*

LUNGS.—Several adhefions were found between the pleura coftalis and the pleura pulmonalis; and in each fide of the lungs a large ulcer was difcovered, containing a confiderable quantity of pus. The whole texture of the lungs was very much difeafed. The purulent matter did not appear to have found its way through the trachea.

HEART.—There was very little fat about the heart; but it was furrounded with a remarkable quantity of a peculiar gelatinous matter. In other refpects, it appeared quite natural.

STOMACH.—The ftomach was uncommonly fmall. The mufcular coat appeared a good deal thicker than ufual; and alfo whiter. It contained only a fmall quantity of a yellow-greenifh, gelatinous matter, the chemical properties of which, unluckily, were not afcertained.

COLON.—The colon was likewife very much contracted in its fize, and fully as much fo in proportion as the ftomach. The inteftines were empty, and in general of a fmall fize.

MESENTERY.—The whole of the mefentery was very much difeafed. All the glands were remarkably enlarged; fome of them very hard and of irregular texture; fome others fofter and of an uniform fpherical fhape. Many of the lacteals could be feen confiderably *enlarged.*

LIVER.—The liver appeared quite found, and natural in every refpect.

PANCREAS.—The pancreas was of a paler colour and of a harder confiftence than common.

The SPLEEN—was quite natural.

Ureters.—There was nothing unufual in the ureters, except that they appeared fomewhat whiter than common.

Kidneys.—The right kidney was of a natural fize; the left was rather larger than ufual. Both of them had externally a natural appearance; but on being cut through, the cortical fubftance appeared uncommonly vafcular; and the fubftance of the tubuli uriniferi & proceffus mamillaris was whiter and more tender than ufual. The left kidney was taken away for the fake of a more minute examination.

Bladder.—The bladder was rather larger than common, and diftended with a quantity of muddy urine.

Penis.—A fmall quantity of mucous or purulent matter was found oozing from the orifice of the urethra, which appeared flightly inflamed; and fome contractions were perceived in it.

Glands.—All the glandular fyftem, and efpecially the glands in the neck and groin, were very hard and confiderably enlarged. Thofe of the mefentery, as already obferved, were remarkably difeafed.

Lymphatics.—The *lymphatic veffels* alfo were generally enlarged; but this was more particularly obvious in the inteftines. The ftate of the lymphatics in the lungs could not be diftinctly afcertained.

Urine.—Some urine was taken out of the bladder after death, which being evaporated, yielded a refiduum which had a very ftrong urinous fmell, and did not appear to contain any faccharine matter: at leaft the prefence of fugar was not difcoverable by the fenfes.

N. B. The left kidney having been diffected and kept in fpirits for a few days, exhibited the appearances above defcribed, except that the proceffus mamillaris had loft that fhining white colour which was remarked on the firft infpection.

I had collected fome blood from one of the large veins, but finding no fugar in the urine, I did not think it neceffary to fubmit the blood to any chemical examination.

This diffection, I am afraid, will be found to throw but little additional light upon the theory of the difeafe; yet it may, I believe, by exhibiting a variety of morbid appearances, which had not been obferved in former inftances of the fame difeafe, and a found ftate of feveral organs which, in other cafes had been found altered, tend to corroborate the opinion that diabetes does not originaly depend upon any organic difeafe in the abdominal vifcera, but rather upon fome change in the procefs of digeftion and affimiassimilation, in consequence of which, after a length of time, some of the organs connected with those functions, may become more or less diseased. The state of the mesentery, in this case, corresponded in a general point of view, with the theory which you and Dr. RUTHERFORD have so ingeniously developed; but at the same time it must be acknowledged, that the morbid appearances alluded to were by no means exclusively in the mesenteric system.

I will not fatigue you with any farther speculations upon this curious and still problematical disease. Permit me only to recal to your attention the curious combination of diabetic and phthisical symptoms, which occured in the present case. The skin continued always dry, and not only the colliquative sweats, but all perspiration whatever was totally prevented. On the other hand, the colliquative diarrhœa took place in a very high degree and the obstinate costiveness which is peculiar to diabetes, entirely disappeared at the latter end of the disorder.

I have the honor to be,

Dear Sir,

CAMOMILE-STREET, Your humble, obedient servant,

*London, July 1st,* 1799. A. MARCET.

P. S. I have read the above account to Dr. MARSHALL, Dr. WILLAN, and Dr. DELARIVE, all of whom, I am happy to find, agree with me in their recollection of the circumstances therein mentioned.

*To Dr. Rollo, Surgeon-General,*
*Royal Artillery, Woolwich.*

